# Factors associated with death among hospitalized older adults with COVID-19 in Brazil*

**DOI:** 10.1590/1518-8345.8031.4945

**Published:** 2026-10-16

**Authors:** Luiz Hiroshi Inoue, Wanessa Cristina Baccon, Francielle Renata Danielli Martins Marques, Maria Aparecida Salci, Vanessa Denardi Antoniassi Baldissera, Stéfane Lele Rossoni, Lígia Carreira

**Affiliations:** 1 Universidade Estadual de Maringá, Maringá, PR, Brazil.

**Keywords:** Aged, Mortality, Hospitalization, COVID-19, Nursing, Risk Factors

## Abstract

**Objective:**

to analyze the factors associated with death among hospitalized older adults with COVID-19 in Brazil.

**Method:**

a cross-sectional study conducted using publicly available secondary data from hospitalized older adults who died from COVID-19. Odds ratios were obtained through the adjustment of a logistic regression model.

**Results:**

the analysis comprised 468,239 deaths of hospitalized older adults in Brazil. Some factors were associated with death, such as: age between 75 and 84 years (OR=1.77; 95% CI=1.74-1.79) and 85 years or older (OR=3.01; 95% CI=2.96-3.06); race/ethnicity White (OR=1.11; 95% CI=1.05-1.17), Indigenous (OR=1.54; 95% CI=1.34-1.76), Brown (OR=1.28; 95% CI=1.22-1.35), and Black (OR=1.48; 95% CI=1.39-1.57); male sex (OR=1.27; 95% CI=1.25-1.28); COVID-19 vaccination (OR=0.68; 95% CI=0.67-0.69); use of invasive ventilatory support (OR=19.54; 95% CI=19.14-19.95) and non-invasive ventilatory support (OR=1.78; 95% CI=1.75-1.80).

**Conclusion:**

an association was identified between age, sex, race/ethnicity, COVID-19 vaccination, and the use of ventilatory support with COVID-19-related death.

## Highlights

**(1)** Factors associated with COVID-19-related death among hospitalized older adults.

**(2)** Conditions associated with higher odds of death from COVID-19 among older adults.

**(3)** Effectiveness of COVID-19 vaccination in older adults as a preventive strategy.

## Introduction

The coronavirus disease 2019 (COVID-19) pandemic caused thousands of deaths worldwide, and mortality among high-risk populations remains widely discussed in the literature as a way to promote preventive measures and health improvements for these groups^1^. Due to its high transmissibility, within less than six months of the pandemic the disease had already claimed more than one million lives^2^, accumulating more than seven million deaths worldwide^3^. In Brazil, data from February 2025 indicate that the number of fatalities due to COVID-19 has reached nearly 715,000 deaths^4^. 

Older adults were the most affected population in terms of COVID-19 mortality, presenting higher odds of death compared with younger individuals^5^. Advanced age and the diagnosis of pre-existing diseases are factors associated with worse COVID-19 outcomes^6^. 

The literature identifies important predictors of disease complications, including genetic and environmental markers, immunosuppression, cardiovascular, respiratory, and gastrointestinal diseases, place of residence, and Intensive Care Unit (ICU) admission^7^. Thus, older adults represent a high-risk group due to factors related to the aging process, such as physiological changes and multiple comorbidities, making this population more susceptible to infection and progression to death^8^. 

Analyzing the impact of COVID-19 on the older population enables the identification of factors associated with mortality, as well as the understanding of clinical manifestations that may compromise quality of life and lead to short- and long-term health complications^9^. This investigation not only contributes to advancing scientific knowledge about the disease but also provides essential support for the development of public health strategies targeted at this age group.

Understanding the determinants that influence the clinical outcomes of COVID-19 among older adults may enable health managers to improve public policies that prioritize the continuous monitoring of this population, recognized as particularly vulnerable during the pandemic. In this context, given the intrinsic vulnerability of older adults to severe disease outcomes, it was hypothesized that sociodemographic and clinical variables may be associated with COVID-19-related death among older adults. Therefore, the aim of this study was to analyze the factors associated with death among hospitalized older adults with COVID-19 in Brazil.

## Method

### Study design

This is a cross-sectional study designed based on publicly available secondary data, addressing deaths among hospitalized older adults due to COVID-19. The study followed the criteria established by the Strengthening the Reporting of Observational Studies in Epidemiology (STROBE) guidelines, ensuring methodological rigor and transparency in the presentation of the findings^10^.

The present study is part of a multicenter research project conducted in partnership between the *Universidade Estadual de Maringá* (UEM) and the Health Department of the State of Paraná (SESA/PR), with funding from the *Conselho Nacional de Desenvolvimento Científico e Tecnológico* (CNPq).

### Setting

The study was conducted nationwide, covering the entire Brazilian territory. According to estimates from the *Instituto Brasileiro de Geografia e Estatística* (IBGE) for the year 2022, the country’s population is approximately 203 million people, of which 32,113,490 belong to the older adult age group^11^.

### Population

During the analyzed period, 1,070,336 cases of older adults diagnosed with Severe Acute Respiratory Syndrome (SARS) were identified across the Brazilian territory between March 2020 and March 2023. 

### Sample definition

Individuals classified as older adults were selected, defined as those aged 60 years or older, in accordance with Article 1 of the Statute of the Older Adult (Law nº 10,741/2003) - Brazil, who developed SARS and were hospitalized with COVID-19.

### Study variables

The dependent variable referred to the patient outcome recorded in the notification system, classified as death due to COVID-19 or hospital discharge/death from other causes. This classification was determined based on the analysis of the specific field indicated in the corresponding notification form.

The independent variables were divided into two main groups: sociodemographic variables and clinical variables. The sociodemographic characteristics included: age group (60-74; 75-84; 85 years or older), sex (female; male), race/ethnicity (White; Black; Asian; Indigenous; Brown), education level (Elementary School I; Elementary School II; High School; with no formal education; Higher Education), and area of residence (peri-urban; rural; urban).

Clinical variables included symptoms such as: fever (yes; no), cough (yes; no), sore throat (yes; no), dyspnea (yes; no), respiratory distress (yes; no), oxygen saturation < 95% (yes; no), diarrhea (yes; no), vomiting (yes; no), abdominal pain (yes; no), fatigue (yes; no), loss of smell (yes; no), and loss of taste (yes; no). They also included comorbidities such as: heart disease (yes; no), hematological disease (yes; no), Down syndrome (yes; no), liver disease (yes; no), asthma (yes; no), diabetes (yes; no), neurological disease (yes; no), chronic lung disease (yes; no), immunosuppression (yes; no), kidney disease (yes; no), and obesity (yes; no). In addition, other factors were considered, such as COVID-19 vaccination (yes; no), ICU admission (yes; no), and use of ventilatory support (none; invasive; non-invasive).

### Data collection

The information was extracted from individual case report forms of SARS available on the SIVEP-*Gripe* platform, an epidemiological surveillance system linked to the Health Surveillance Secretariat (SVS, in Portuguese) for monitoring cases and deaths from SARS^12^. The analyzed period comprised March 2020 to March 2023, selected in order to include the most up-to-date data available at the time of collection.

### Data processing and analysis

The data were organized in electronic spreadsheets and processed using the statistical software R Core Team (2024), version 4.4.2. For descriptive analysis, absolute and relative frequencies of categorical variables were calculated according to the outcome, using the Pearson’s chi-square test for comparisons. For numerical variables, summary measures (minimum, mean, maximum, and standard deviation) were calculated.

The comparison tests used to verify significant differences between groups that died and those that did not were Student’s t-test, Mann-Whitney test, Kruskal-Wallis test, and ANOVA. 

The analysis of factors associated with the outcome of death due to COVID-19 was performed using a binomial logistic regression model. The value 1 was assigned to death due to COVID-19 and 0 to discharge or death from other causes. The level of significance adopted in this analysis was 5%, with a 95% confidence interval.

### Ethical considerations

As this study used publicly available secondary data, approval by a Research Ethics Committee was waived. However, the multicenter study to which the present research is linked received approval from the Research Ethics Committee of the *Universidade Estadual de Maringá*, under approval nº 4,165,272 and CAAE: 34787020.0.0000.0104. In addition, it received approval from the Health Department of the State of Paraná, represented by the *Hospital do Trabalhador*, under approval nº 4,214,589 and CAAE: 34787020.0.3001.5225, on August 15, 2020. 

## Results

A total of 1,064,033 records of hospitalized older adults with a confirmed diagnosis of SARS due to COVID-19 were analyzed. Among these, 468,237 cases progressed to death due to the disease, while 536,997 were discharged from hospital. The minimum, mean, and maximum ages observed among confirmed COVID-19 cases that resulted in death were 60, 75.3, and 104 years, respectively, with a standard deviation of 9.55.

A gradual increase in the percentage of deaths was observed with increasing age. Although the percentage of deaths from COVID-19 in the 60-74 age group (41%) was lower compared with those who were discharged from hospital (58.9%), this pattern was reversed in the 75-84 age group (51%) and among those 85 years or older (58.2%), when compared with individuals in the same age groups who were discharged from hospital. Regarding race/ethnicity, a higher percentage of deaths from COVID-19 was observed among older adults who self-identified as Black (53.1%) and Indigenous (52.3%) (Table 1). 

**Table 1:** Sociodemographic and clinical characteristics of hospitalized older adults with SARS* who died from COVID-19 in Brazil (n = 468,237). Brazil, 2023

Variables	Case outcome						p-value^†^
	Hospitalized		Deaths		Hospital discharge		
	(n = 1,064,033)		(n = 468,237)		(n = 536,997)		
	n^‡^	%^§^	n^‡^	%^§^	n^‡^	%^§^	
Sociodemographic variables							
Age group							
60 to 74 years	570869	56.8	234386	41.1	336483	58.9	<0.001
75 to 84 years	278597	27.7	143150	51.4	135447	48.6	<0.001
85 years or older	155768	15.5	90701	58.2	65067	41.8	<0.001
Race/ethnicity							
Asian	10568	1.3	4714	44.6	5854	55.4	<0.001
White	456824	55.1	211448	46.3	245376	53.7	<0.001
Indigenous	1766	0.2	923	52.3	843	47.7	0.06
Brown	317314	38.3	156533	49.3	160781	50.7	<0.001
Black	42909	5.2	22800	53.1	20109	46.9	<0.001
Sex							
Female	482487	48.0	214093	44.4	268394	55.6	<0.001
Male	522646	52.0	254099	48.6	268547	51.4	<0.001
Education level							
Elementary School I	136204	39.2	70381	51.7	65823	48.3	<0.001
Elementary School II	66333	19.1	33342	50.3	32991	49.7	0.17
High School	70897	20.4	34385	48.5	36512	51.5	<0.001
With no formal education	38603	11.1	22067	57.2	16536	42.8	<0.001
Higher Education	35239	10.1	15774	44.8	19465	55.2	<0.001
Area of residence							
Peri-urban	3043	0.3	1577	51.8	1466	48.2	0.04
Rural	47792	5.4	22764	47.6	25028	52.4	<0.001
Urban	837953	94.3	388237	46.3	449716	53.7	<0.001
Clinical variables							
Fever							
No	309459	38.1	140075	45.3	169384	54.7	<0.001
Yes	502416	61.9	229594	45.7	272822	54.3	<0.001
Cough							
No	199616	23.3	98491	49.3	101125	50.7	<0.001
Yes	656076	76.7	287337	43.8	368739	56.2	<0.001
Sore throat							
No	538834	78.8	245546	45.6	293288	54.4	<0.001
Yes	145360	21.2	61507	42.3	83853	57.7	<0.001
Dyspnea							
No	169020	19.4	58041	34.3	110979	65.7	<0.001
Yes	701491	80.6	349247	49.8	352244	50.2	<0.001
Respiratory distress							
No	242232	30.2	87892	36.3	154340	63.7	<0.001
Yes	560223	69.8	288193	51.4	272030	48.6	<0.001
Oxygen saturation < 95%							
No	185602	22.1	65109	35.1	120493	64.9	<0.001
Yes	654810	77.9	327136	50.0	327674	50.0	<0.001
Diarrhea							
No	563198	83.2	256225	45.5	306973	54.5	<0.001
Yes	113625	16.8	47791	42.1	65834	57.9	<0.001
Vomiting							
No	592319	89.2	268438	45.3	323881	54.7	<0.001
Yes	71840	10.8	30121	41.9	41719	58.1	<0.001
Abdominal pain							
No	525005	91.9	232288	44.2	292717	55.8	<0.001
Yes	46073	8.1	19356	42.0	26717	58.0	<0.001
Fadiga							
No	397366	65.2	175839	44.3	221527	55.7	<0.001
Yes	212032	34.8	92916	43.8	119116	56.2	<0.001
Loss of smell							
No	512583	89.5	228190	44.5	284393	55.5	<0.001
Yes	60133	10.5	22956	38.2	37177	61.8	<0.001
Loss of taste							
No	509614	89.0	227375	44.6	282239	55.4	<0.001
Yes	62786	11.0	23474	37.4	39312	62.6	<0.001
Heart disease							
No	119464	21.0	80152	67.1	39312	32.9	<0.001
Yes	449231	79.0	216109	48.1	233122	51.9	<0.001
Hematological disease							
No	441456	98.3	206166	46.7	235290	53.3	<0.001
Yes	7795	1.7	4180	53.6	3615	46.4	<0.001
Down syndrome							
No	446779	99.6	209055	46.8	237724	53.2	<0.001
Yes	1594	0.4	755	47.4	839	52.6	0.04
Liver disease							
No	439323	98.1	204832	46.6	234491	53.4	<0.001
Yes	8686	1.9	5136	59.1	3550	40.9	<0.001
Asthma							
No	430936	95.3	202260	46.9	228676	53.1	<0.001
Yes	21168	4.7	8841	41.8	12327	58.2	<0.001
Diabetes							
No	260000	45.6	120059	46.2	139941	53.8	<0.001
Yes	310429	54.4	150709	48.5	159720	51.5	<0.001
Neurological disease							
No	409022	87.7	187737	45.9	221285	54.1	<0.001
Yes	57534	12.3	32808	57.0	24726	43.0	<0.001
Chronic lung disease							
No	411704	88.5	189910	46.1	221794	53.9	<0.001
Yes	53559	11.5	29607	55.3	23952	44.7	<0.001
Immunosuppression							
No	427735	94.5	198615	46.4	229120	53.6	<0.001
Yes	24879	5.5	13890	55.8	10989	44.2	<0.001
Kidney disease							
No	414401	89.7	189995	45.8	224406	54.2	<0.001
Yes	47703	10.3	28836	60.4	18867	39.6	<0.001
Obesity							
No	399785	87.4	185499	46.4	214286	53.6	<0.001
Yes	57889	12.6	29698	51.3	28191	48.7	<0.001
COVID-19 vaccination							
No	145596	34.4	73067	50.2	72529	49.8	0.16
Yes	277117	65.6	110663	39.9	166454	60.1	<0.001
ICU^||^ admission							
No	504578	57.8	149529	29.6	355049	70.4	<0.001
Yes	368945	42.2	247501	67.1	121444	32.9	<0.001

*SARS = Severe Acute Respiratory Syndrome; ^†^p-value = Statistical measure indicating the probability that the observed results occurred by chance, assuming the null hypothesis (*H0*) is true; ^‡^n = Sample size; ^§^% = Percentage; ^||^ICU = Intensive Care Unit

The percentage of deaths from COVID-19 among men (48.6%) was lower when compared with the group that was discharged from hospital (51.4%). A similar pattern was observed among women, in whom the percentage of deaths from COVID-19 was lower (44.4%) compared with those discharged from hospital (55.6%). 

When education level was analyzed, the group with higher education presented the lowest percentage of death from COVID-19 (44.8%). In contrast, among older adults with no formal education, this pattern was reversed, with a higher percentage of deaths from COVID-19 (57.1%). 

Regarding area of residence, a higher percentage of deaths from COVID-19 was observed among older adults living in peri-urban areas (51.8%). 

With regard to clinical characteristics, the analyses showed higher percentages of deaths from COVID-19 among older adults presenting respiratory distress (51.4%), hematological disease (53.6%), liver disease (59.1%), neurological disease (57%), chronic lung disease (55.2%), immunosuppression (55.8%), kidney disease (60.4%), obesity (51.3%), ICU admission (67.1%), and use of invasive ventilatory support (85.4%).

Table 2 presents the results of the Kruskal-Wallis test used to compare medians between the groups that progressed to death due to COVID-19 and those who did not, for the variables age and length of hospital stay. The test indicates significant differences in the medians for both variables, showing that older adults who died from COVID-19 had a higher median age and a longer length of hospital stay compared with those who were discharged or died from other causes.

**Table 2:** Kruskal-Wallis test for comparison of medians - Death due to COVID-19 vs. Discharge/Death from other causes. Brazil, 2023

Variable	Outcome	Median	p-value*
Age	Discharge/Death from other causes	71	<0.001
	Death due to COVID-19	74	
Length of hospital stay (days)	Discharge/Death from other causes	7	<0.001
	Death due to COVID-19	9	

*p-value = statistical measure indicating the probability that the observed results occurred by chance, assuming the null hypothesis (*H0*) is true

Figure 1 presents the temporal trend of the monthly death rates among the older adults included in this study. From this figure, it is possible to observe that shortly after the beginning of the vaccination campaign there appears to be a change in the behavior of the mortality rate among the individuals analyzed.

**Figure 1:**
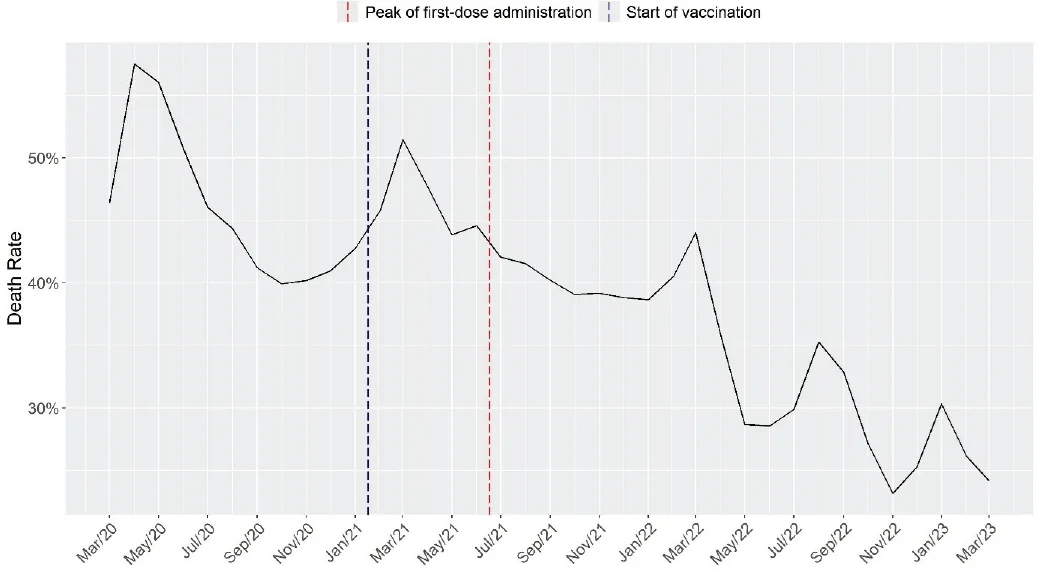
Death rate among older adults with COVID-19 between March 2020 and March 2023

However, in order to verify the trends in this time series, as well as to estimate inflection points (where a change in the pattern occurs) and compare pre- and post-vaccination trends, generalized fluctuation tests were applied to estimate the breakpoints, the Mann-Kendall test was used to evaluate trends, and the Chow test was applied to compare the different periods of the series.

According to the algorithm used to estimate the trend change point (breakpoint), it was observed that the number of breakpoints required to explain the series, while reducing the Akaike Information Criterion (AIC) and the Residual Sum of Squares (RSS), was one breakpoint, located in January 2021, coinciding with the beginning of the vaccination campaign.

The series was divided into two segments, considering the start of vaccination as the cut-off point (which coincided with the estimated breakpoint). The Mann-Kendall test estimated that before the vaccination period the value of τ, corresponding to the slope of the series, was -0.64, and after the start of vaccination the value of τ became -0.78, both statistically significant. These results indicate that in both periods the death rate among older adults showed a decreasing trend; however, the decline became more pronounced after the beginning of vaccination. The Chow test, which compares the series across the two periods, identified significant differences in the behavior of mortality rates before and after the start of vaccination.

In the univariate analyses, among the 31 variables investigated, five were associated with the outcome death due to COVID-19, and these were subsequently included in the adjusted regression model (Table 3).

**Table 3:** Adjusted model of the associations between sociodemographic and clinical characteristics of older adults who died from COVID-19 in Brazil (n = 468,237). Brazil, 2023

Variables	OR*	CI^†^		p-value^‡^
Sociodemographic variables				
Age group				
60 to 74 years	1.00			
75 to 84 years	1.77	1.74	1.79	<0.001
85 years or older	3.01	2.96	3.06	<0.001
Race/ethnicity				
Asian	1.00			
White	1.11	1.05	1.17	<0.001
Indigenous	1.54	1.34	1.76	<0.001
Brown	1.28	1.22	1.35	<0.001
Black	1.48	1.39	1.57	<0.001
Sex				
Female	1.00			
Male	1.27	1.25	1.28	<0.001
Clinical variables				
COVID-19 vaccination				
No	1.00			
Yes	0.68	0.67	0.69	<0.001
Ventilatory support				
None	1.00			
Invasive	19.54	19.14	19.95	<0.001
Non-invasive	1.78	1.75	1.80	<0.001

*OR = Odds Ratio; ^†^CI = Confidence Interval; ^‡^p-value = statistical measure indicating the probability that the observed results occurred by chance, assuming the null hypothesis (*H0*) is true

The 75-84 age group was associated with death due to COVID-19, with 77% higher odds of death from disease complications compared with older adults aged 60-74 years. In the same comparison, individuals aged 85 years or older had three times higher odds of death due to COVID-19. 

Regarding race/ethnicity, higher odds of death due to COVID-19 were observed among older adults who self-identified as Indigenous (54%) and Black (48%), when compared with those classified as Asian. 

In addition, older men presented 1.27 times higher odds of death due to COVID-19 compared with older women. Older adults who received COVID-19 vaccination had a 32% reduction in the odds of death compared with those who were not vaccinated. 

Regarding the use of ventilatory support, the use of this intervention was associated with COVID-19-related death, with markedly higher odds of death among older adults who required invasive ventilatory support (19.54 times) and non-invasive ventilatory support (1.78 times) compared with hospitalized individuals who did not require ventilatory support.

## Discussion

This study aimed to analyze the factors associated with death among hospitalized older adults with COVID-19 in Brazil. The findings showed that the likelihood of older adults progressing to death due to COVID-19 increases depending on certain factors to which individuals are exposed. Advanced age, combined with clinical factors, represents a negative prognosis and a higher risk of death from COVID-19 in this population^6^. 

Findings from a study conducted in Iran with 1,694 older adults demonstrated that age is a predictive factor associated with death due to COVID-19^13^. Being 75 years or older increased the odds of death from the disease by more than two times compared with the 60-74 age group. In addition to age, the study showed that factors such as the hospitalization unit and laboratory indicators were the main predictors of death due to COVID-19^13^.

Recent studies highlight the exacerbated effects and increased mortality from COVID-19 among older adults associated with cellular senescence, a functional alteration related to aging that is considered a central factor in the hyperinflammatory response triggered by SARS-CoV-2^14^ ^)-(^ ^16^. The virus is capable of inducing cellular senescence, intensifying the senescence-associated secretory phenotype, which mainly consists of pro-inflammatory molecules, extracellular matrix-degrading enzymes, complement system activators, and pro-coagulant substances released by senescent cells^15^. These effects are particularly aggravated in older adults due to the greater presence of senescent cells in their tissues. This process triggers cytokine storms, infiltration of immune cells that damage tissues, endothelial inflammation, fibrosis, and microthrombus formation, which contribute to the failure of vital organs and death^14^.

It is important to emphasize that the association between advanced age and the outcome of death goes beyond cellular alterations. Although the analyses in this study did not identify an association between pre-existing diseases and death among older adults with COVID-19, the literature reports important associations between pre-existing comorbidities and an increased risk of death from the disease^7^. The likelihood of death from COVID-19 increases in individuals with cardiovascular, renal, metabolic, oncological, and respiratory diseases, such as Chronic Obstructive Pulmonary Disease (COPD)^6^ ^),(^ ^17^, which was considered the third leading cause of death worldwide, according to the World Health Organization ranking, in 2021^18^. 

Another important factor associated with a higher likelihood of death from COVID-19 is sex. Men aged 60 years or older are more likely to die from the disease when compared with women for several reasons. Advanced age combined with environmental and behavioral factors, such as smoking, increases men’s exposure to risk. Moreover, the higher susceptibility of men to complications and death from COVID-19 may also be attributed to differences in sex hormones associated with inflammatory processes and to the reduced expression of angiotensin-converting enzyme 2 (ACE2), which may protect the lungs from acute injury caused by viral infections^19^. 

Regarding race/ethnicity, findings from a study conducted with Indigenous populations in the state of Amapá, Brazil, showed that advanced age combined with cardiovascular diseases increases the odds of death from COVID-19 by up to four times. Furthermore, older adults residing in metropolitan regions had 2.90 times higher odds of death from COVID-19 when compared with Indigenous individuals living in inland regions of the state^20^. 

Older adults who self-identify as Black face greater vulnerability to infection and death from COVID-19 due to social challenges such as low income, barriers to accessing health services, limited knowledge about the disease, dissemination of misinformation, lack of culturally sensitive public policies, racial discrimination within health systems, and vaccine hesitancy regarding COVID-19 vaccines^21^. In addition, studies indicate that the combination of advanced age and belonging to the Black racial group is strongly associated with a higher risk of hospitalization, admission to intensive care units, clinical complications, and death from the disease^22^.

Regarding COVID-19 vaccination, findings from a meta-analysis study indicate a protective effect among older adults who received the vaccine. In an analysis including more than 3 million participants, vaccinated older adults showed a reduction of more than 80% in the risk of infection and death from COVID-19^23^. Furthermore, recent studies have emphasized that the protection conferred by vaccination in this group should be interpreted in light of two complementary phenomena: immunosenescence and the temporal decline in vaccine effectiveness, particularly for outcomes such as infection and symptomatic disease, with relatively greater preservation for severe outcomes. In this regard, an international meta-analysis demonstrated that effectiveness tends to decline progressively over the months and that booster doses restore higher levels of protection, a factor particularly relevant for older adults, in whom the immune response is typically less robust and more heterogeneous^24^. 

In line with these findings, analyses conducted in healthcare networks and vaccine effectiveness surveillance in real-world settings have demonstrated an additional benefit of updated vaccines against recent variants, with a more pronounced impact among older age groups. Estimates of vaccine effectiveness in older adults indicate a significant reduction in hospitalization following recent seasonal vaccination, reinforcing the need for booster strategies targeted at older adults and vulnerable groups, including scenarios characterized by the predominance of Omicron sublineages genetically divergent from previous vaccine compositions^25^ ^)-(^ ^27^. 

Regarding the use of ventilatory support, hospitalized older adults with COVID-19 and admitted to the ICU have a higher likelihood of death when this intervention is required. A study involving 1,994 participants showed that individuals who required non-invasive and invasive ventilatory support had 2.25 and 24 times higher odds of death, respectively, compared with those who did not require this intervention^28^. 

It is also important to highlight that, beyond the intrinsic severity of the clinical condition, the trajectory of escalation in ventilatory support may influence patient outcomes. Among patients with acute respiratory failure due to COVID-19, the use of non-invasive strategies, such as high-flow nasal cannula and non-invasive ventilation, has been associated with benefits in selected subgroups. However, failure of these modalities, particularly when accompanied by delayed intubation, may be associated with worse prognosis^29^. Multicenter studies have demonstrated higher in-hospital mortality among patients undergoing late intubation compared with early intubation, emphasizing the importance of close monitoring, objective criteria for treatment failure, and timely clinical decision-making, especially among older adults, whose physiological reserve is typically reduced^30^. 

Furthermore, invasive mechanical ventilation in this specific group is frequently associated with adverse outcomes related to ICU admission, including delirium, acquired weakness, prolonged sedation, and infectious complications, particularly ventilator-associated pneumonia, which may affect both length of hospital stay and mortality^31^ ^)-(^ ^32^. Studies involving older adults in more advanced age groups also report poorer outcomes when non-invasive ventilation is required during pandemic scenarios, suggesting that advanced age, frailty, and respiratory severity tend to interact as determinants of worse clinical progression^33^. 

In the present study, some limitations inherent to the use of secondary data derived from administrative health systems should be acknowledged, as such sources may contain underreporting or incomplete information in certain variables. Nevertheless, the analytical methodology adopted was carefully selected to minimize the impact of missing data on the analyses performed, ensuring greater rigor and reliability in the health-related inferences drawn. The statistical results that did not identify an association between pre-existing comorbidities and death due to COVID-19 in this study may be related to the absence of standardized analytical methods for research of this nature.

Despite these limitations, the results obtained allowed for a comprehensive analysis of the epidemiological and clinical characteristics of COVID-19 infection and mortality among older adults in Brazil. Furthermore, the role of studies with this methodological design is reinforced as a driver of similar investigations in other settings, expanding the understanding of contextual determinants and the behavior of the disease across different population strata. This approach highlights the relevance of scientific production oriented toward practical application and the generation of tangible societal benefits.

In this context, the findings of the present study provide strategic support for health managers, offering scientific evidence for the formulation, strengthening, and implementation of public health policies that reflect the realities experienced by the older population. By considering existing vulnerabilities, such policies may promote more effective and targeted interventions, ensuring positive impacts on health actions while strengthening equity and the quality of healthcare provided.

## Conclusion

The factors associated with death among hospitalized older adults with COVID-19 were related to age, sex, race/ethnicity, COVID-19 vaccination, and the use of ventilatory support. Additionally, being 80 years or older, male, of Indigenous ethnicity, and requiring invasive ventilatory support were conditions associated with the highest odds of death.

The administration of the COVID-19 vaccine proved effective in providing protection to older adults exposed to SARS-CoV-2, highlighting the importance of preventive strategies such as immunization in minimizing the adverse impacts of the pandemic on a more vulnerable population group. This relationship underscores the need to maintain and strengthen vaccination campaigns as a public health strategy in order to reduce the multisystem repercussions of COVID-19 on the life, health, and well-being of older adults.

The importance of advancing health monitoring strategies for older adults should also be emphasized, particularly those aimed at expanding immunoprevention and reducing misinformation surrounding COVID-19 vaccines. It is expected that the present study will contribute as a foundation for the development of initiatives capable of fully addressing the specific needs of the older population. Among these initiatives, strengthening primary health care actions stands out, aiming to ensure timely access to health services and continuous care, guided by the individual needs of older adults and seeking to mitigate the vulnerabilities of this age group in the context of COVID-19.

## Data Availability

All data generated or analysed during this study are included in this published article.
