# Supplementary material for: Factors associated with death among hospitalized older adults with COVID-19 in Brazil
Source: Rev Lat Am Enfermagem. 2026 Oct 16;34:e4945. doi: 10.1590/1518-8345.8031.4945 (PMC13674978; doi:10.1590/1518-8345.8031.4945)
Supplement: Translation

# Translation: Fatores associados ao óbito de pessoas idosas hospitalizadas por COVID-19 no Brasil*

**DOI:** 10.1590/1518-8345.8031.4946

**Authors:** Luiz Hiroshi Inoue, Wanessa Cristina Baccon, Francielle Renata Danielli Martins Marques, Maria Aparecida Salci, Vanessa Denardi Antoniassi Baldissera, Stéfane Lele Rossoni, Lígia Carreira

**Affiliations:** 1 Universidade Estadual de Maringá́, Maringá, PR, Brasil.

**Keywords:** Idosos, Mortalidade, Hospitalização, COVID-19, Enfermagem, Fatores de Risco

## Abstract

**Objetivo:**

analisar os fatores associados ao óbito de pessoas idosas hospitalizadas por COVID-19 no Brasil.

**Método:**

estudo transversal conduzido com dados secundários de domínio público de pessoas idosas hospitalizadas que evoluíram a óbito por COVID-19. As razões de chances foram obtidas por meio do ajuste de modelo de regressão logística.

**Resultados:**

a análise foi composta por 468.239 óbitos de pessoas idosas hospitalizadas no Brasil. Alguns fatores foram associados ao óbito, tais como: idade entre 75 e 84 anos (OR=1,77; IC_95%_=1,74-1,79) e 85 anos ou mais (OR=3,01; IC_95%_=2,96-3,06); raça/cor branca (OR=1,11; IC_95%_=1,05-1,17), indígena (OR=1,54; IC_95%_=1,34-1,76), parda (OR=1,28; IC_95%_=1,22-1,35) e preta (OR=1,48; IC_95%_=1,39-1,57); sexo masculino (OR=1,27; IC_95%_=1,25-1,28); vacina contra a COVID-19 (OR=0,68; IC_95%_=0,67-0,69); uso de suporte ventilatório invasivo (OR=19,54; IC_95%_=19,14-19,95) e não invasivo (OR=1,78; IC_95%_=1,75-1,80).

**Conclusão:**

identificou-se associação entre idade, sexo, raça/cor, vacina contra a COVID-19 e uso de suporte ventilatório com o óbito por COVID-19.

## Destaques

**(1)** Fatores associados ao óbito por COVID-19 em pessoas idosas hospitalizadas.

**(2)** Condições associadas a maiores chances de óbito por COVID-19 em pessoas idosas.

**(3)** Eficácia da vacina contra a COVID-19 em pessoas idosas como estratégia preventiva.

## Introdução

A pandemia de *coronavirus disease* 2019 (COVID-19) causou milhares de óbitos em âmbito mundial, e a mortalidade em populações de risco ainda é amplamente discutida na literatura como forma de promover medidas de prevenção e melhorias em saúde para esses grupos^1^. Com alto poder de disseminação, em menos de seis meses de pandemia, a doença já havia ceifado mais de um milhão de vidas^2^, acumulando mais de sete milhões de óbitos em todo o mundo^3^. No Brasil, dados de fevereiro de 2025 apontam que o número de vítimas fatais da COVID-19 chega a quase 715 mil óbitos^4^. 

A população idosa foi a mais afetada quanto à mortalidade por COVID-19, apresentando maiores chances de óbito quando comparada a pessoas mais jovens^5^. A idade avançada e o diagnóstico de doenças pré-existentes são fatores associados aos piores desfechos da COVID-19^6^. 

A literatura apresenta importantes preditores de complicações da doença, como marcadores genéticos e ambientais, imunossupressão, doenças cardiovasculares, respiratórias e gastrintestinais, local de residência e admissão em Unidade de Terapia Intensiva (UTI)^7^. Assim, o grupo das pessoas idosas enquadra-se em um nível de alto risco, devido a fatores relacionados ao processo de envelhecimento, como alterações fisiológicas e múltiplas comorbidades, tornando essa população mais suscetível a contrair a doença e evoluir para o óbito^8^. 

A análise do impacto da COVID-19 na população idosa possibilita a identificação de fatores associados à mortalidade, bem como a compreensão das manifestações clínicas que podem comprometer a qualidade de vida e ocasionar agravos à saúde a curto e longo prazos^9^. Essa investigação não apenas contribui para o avanço do conhecimento científico sobre a doença, mas também oferece subsídios essenciais para a formulação de estratégias de saúde pública direcionadas a esse grupo etário.

Ao compreender os determinantes que influenciam o desfecho clínico da COVID-19 em pessoas idosas, gestores de saúde podem aprimorar políticas públicas que priorizem o monitoramento contínuo dessa população, reconhecida como particularmente vulnerável durante a pandemia. Nesse sentido, dada a vulnerabilidade intrínseca da população idosa a desfechos severos da doença, formulou-se a hipótese de que variáveis sociodemográficas e clínicas podem estar associadas ao óbito de pessoas idosas por COVID-19. Dessa forma, o objetivo do presente estudo foi analisar os fatores associados ao óbito de pessoas idosas hospitalizadas por COVID-19 no Brasil.

## Método

### Tipo de estudo

Trata-se de um estudo transversal delineado com base em dados secundários de acesso público, abordando o óbito de pessoas idosas hospitalizadas em decorrência da COVID-19. A condução da pesquisa seguiu os critérios estabelecidos pela ferramenta *Strengthening the Reporting of Observational Studies in Epidemiology* (STROBE), assegurando rigor metodológico e transparência na apresentação dos achados^10^.

O presente estudo está vinculado a uma pesquisa multicêntrica, realizado em parceria entre a Universidade Estadual de Maringá (UEM) e a Secretaria de Saúde do Estado do Paraná (SESA/PR), com financiamento do Conselho Nacional de Desenvolvimento Científico e Tecnológico (CNPq).

### Local

A pesquisa foi realizada em âmbito nacional, abrangendo todo o território brasileiro. De acordo com estimativas do Instituto Brasileiro de Geografia e Estatística (IBGE) para o ano de 2022, a população do país é composta por aproximadamente 203 milhões de pessoas, das quais 32.113.490 pertencem à faixa etária idosa^11^.

### População

No período analisado, foram observados 1.070.336 casos de pessoas idosas diagnosticadas com Síndrome Respiratória Aguda Grave (SRAG) residentes em todo o território brasileiro, entre março de 2020 e março de 2023. 

### Definição da amostra

Foram selecionados indivíduos considerados pessoas idosas, ou seja, aqueles com idade igual ou superior a 60 anos, conforme disposto no artigo 1º do Estatuto do Idoso (Lei 10.741/2003) - Brasil, que desenvolveram SRAG e foram hospitalizados por COVID-19.

### Variáveis do estudo

A variável dependente refere-se ao desfecho do paciente em relação à notificação, classificada em óbito por COVID-19 ou alta hospitalar/óbito por outras causas. Sua determinação baseou-se na análise do campo específico indicado na ficha de notificação correspondente.

As variáveis independentes foram distribuídas em dois grupos principais: variáveis sociodemográficas e variáveis clínicas. Para as características sociodemográficas foram consideradas: faixa etária (60 a 74; 75 a 84; 85 anos ou mais), sexo (feminino; masculino), raça/cor (branca; preta; amarela; indígena; parda), escolaridade (Ensino Fundamental I; Ensino Fundamental II; Ensino Médio; sem escolaridade; Ensino Superior) e zona de residência (periurbana; rural; urbana).

As variáveis clínicas incluíram sintomas como: febre (sim; não), tosse (sim; não), dor de garganta (sim; não), dispneia (sim; não), desconforto respiratório (sim; não), saturação < 95% (sim; não), diarreia (sim; não), vômito (sim; não), dor abdominal (sim; não), fadiga (sim; não), perda de olfato (sim; não) e perda de paladar (sim; não). Incluíram também comorbidades como: cardiopatia (sim; não), doença hematológica (sim; não), síndrome de Down (sim; não), doença hepática (sim; não), asma (sim; não), diabetes (sim; não), doença neurológica (sim; não), pneumopatia (sim; não), imunodepressão (sim; não), doença renal (sim; não) e obesidade (sim; não). Além disso, foram considerados outros fatores, como: vacina contra a COVID-19 (sim; não), admissão em UTI (sim; não) e uso de suporte ventilatório (não; invasivo; não invasivo).

### Coleta de dados

As informações foram extraídas de fichas de registro individual de casos de SRAG disponibilizadas na plataforma SIVEP-Gripe, instrumento do Sistema de Vigilância Epidemiológica vinculado à Secretaria de Vigilância em Saúde (SVS) para monitoramento de casos e óbitos por SRAG^12^. O período analisado compreendeu março de 2020 a março de 2023, selecionado com o propósito de abranger os dados mais atualizados no momento da coleta.

### Tratamento e análise dos dados

Os dados foram organizados em planilhas eletrônicas e processados no programa estatístico R Core Team (2024), versão 4.4.2. Para a análise descritiva dos dados, foram calculadas as frequências absolutas e relativas das variáveis categóricas em relação ao desfecho, utilizando-se o teste qui-quadrado de Pearson para estabelecer comparações. Para as variáveis numéricas, foram calculadas as medidas-resumo (mínimo, média, máximo e desvio-padrão).

Os testes de comparação realizados para verificar diferenças significativas entre os grupos que evoluíram a óbito ou não foram: teste t de Student, Mann-Whitney, Kruskal-Wallis e ANOVA. 

A análise dos fatores associados ao desfecho de óbito por COVID-19 foi realizada por meio de modelo de regressão logística da família binomial. Considerou-se 1 para o óbito da pessoa idosa por COVID-19 e 0 para alta ou óbito por outras causas. O nível de significância considerado nesta análise foi de 5% e o intervalo de confiança de 95%.

### Aspectos éticos

Por se tratar de um estudo conduzido com dados secundários de domínio público, foi dispensada a análise do Comitê de Ética em Pesquisa. Entretanto, o estudo multicêntrico ao qual a presente pesquisa está vinculada obteve parecer favorável do Comitê de Ética em Pesquisa da Universidade Estadual de Maringá, sob o parecer nº 4.165.272 e CAAE: 34787020.0.0000.0104. Ademais, recebeu aprovação da Secretaria de Saúde do Estado do Paraná, representada pelo Hospital do Trabalhador, obtendo o parecer nº 4.214.589 e CAAE: 34787020.0.3001.5225, em 15 de agosto de 2020. 

## Resultados

Foram analisados 1.064.033 registros de pessoas idosas hospitalizadas com diagnóstico confirmado de SRAG por COVID-19, dos quais 468.237 casos evoluíram a óbito pela doença e 536.997 tiveram alta hospitalar. A idade mínima, média e máxima observada entre os casos confirmados de COVID-19 que evoluíram a óbito foi de 60, 75,3 e 104 anos respectivamente, com um desvio padrão de 9,55.

Observou-se um aumento gradativo no percentual de óbitos conforme o avanço da idade. Embora o percentual de óbitos por COVID-19 na faixa etária de 60 a 74 anos (41%) seja menor quando comparado ao dos que receberam alta hospitalar (58,9%), esse quadro se inverte nas faixas etárias de 75 a 84 anos (51%) e de 85 anos ou mais (58,2%), em comparação com os grupos da mesma faixa etária que receberam alta hospitalar. Em relação à raça/cor, houve uma predominância no percentual de óbitos por COVID-19 de pessoas idosas autodeclaradas pretas (53,1%) e indígenas (52,3%) (Tabela 1). 

**Tabela 1:** Características sociodemográficas e clínicas de pessoas idosas hospitalizadas por SRAG* que evoluíram a óbito por COVID-19 no Brasil (n = 468.237). Brasil, 2023

Variáveis	Evolução do caso						p-valor^†^
	Hospitalizados		Óbitos		Alta hospitalar		
	(n = 1.064.033)		(n = 468.237)		(n = 536.997)		
	n^‡^	%^§^	n^‡^	%^§^	n^‡^	%^§^	
Sociodemográficas							
Faixa etária							
60 a 74 anos	570869	56,8	234386	41,1	336483	58,9	<0,001
75 a 84 anos	278597	27,7	143150	51,4	135447	48,6	<0,001
85 anos ou mais	155768	15,5	90701	58,2	65067	41,8	<0,001
Raça/Cor							
Amarela	10568	1,3	4714	44,6	5854	55,4	<0,001
Branca	456824	55,1	211448	46,3	245376	53,7	<0,001
Indígena	1766	0,2	923	52,3	843	47,7	0,06
Parda	317314	38,3	156533	49,3	160781	50,7	<0,001
Preta	42909	5,2	22800	53,1	20109	46,9	<0,001
Sexo							
Feminino	482487	48,0	214093	44,4	268394	55,6	<0,001
Masculino	522646	52,0	254099	48,6	268547	51,4	<0,001
Escolaridade							
Ensino Fundamental I	136204	39,2	70381	51,7	65823	48,3	<0,001
Ensino Fundamental II	66333	19,1	33342	50,3	32991	49,7	0,17
Ensino Médio	70897	20,4	34385	48,5	36512	51,5	<0,001
Sem escolaridade	38603	11,1	22067	57,2	16536	42,8	<0,001
Superior	35239	10,1	15774	44,8	19465	55,2	<0,001
Zona de Residência							
Periurbana	3043	0,3	1577	51,8	1466	48,2	0,04
Rural	47792	5,4	22764	47,6	25028	52,4	<0,001
Urbana	837953	94,3	388237	46,3	449716	53,7	<0,001
Clínicas							
Febre							
Não	309459	38,1	140075	45,3	169384	54,7	<0,001
Sim	502416	61,9	229594	45,7	272822	54,3	<0,001
Tosse							
Não	199616	23,3	98491	49,3	101125	50,7	<0,001
Sim	656076	76,7	287337	43,8	368739	56,2	<0,001
Dor de garganta							
Não	538834	78,8	245546	45,6	293288	54,4	<0,001
Sim	145360	21,2	61507	42,3	83853	57,7	<0,001
Dispneia							
Não	169020	19,4	58041	34,3	110979	65,7	<0,001
Sim	701491	80,6	349247	49,8	352244	50,2	<0,001
Desconforto respiratório							
Não	242232	30,2	87892	36,3	154340	63,7	<0,001
Sim	560223	69,8	288193	51,4	272030	48,6	<0,001
Saturação < 95%							
Não	185602	22,1	65109	35,1	120493	64,9	<0,001
Sim	654810	77,9	327136	50,0	327674	50,0	<0,001
Diarreia							
Não	563198	83,2	256225	45,5	306973	54,5	<0,001
Sim	113625	16,8	47791	42,1	65834	57,9	<0,001
Vômito							
Não	592319	89,2	268438	45,3	323881	54,7	<0,001
Sim	71840	10,8	30121	41,9	41719	58,1	<0,001
Dor abdominal							
Não	525005	91,9	232288	44,2	292717	55,8	<0,001
Sim	46073	8,1	19356	42,0	26717	58,0	<0,001
Fadiga							
Não	397366	65,2	175839	44,3	221527	55,7	<0,001
Sim	212032	34,8	92916	43,8	119116	56,2	<0,001
Perda de olfato							
Não	512583	89,5	228190	44,5	284393	55,5	<0,001
Sim	60133	10,5	22956	38,2	37177	61,8	<0,001
Perda de paladar							
Não	509614	89,0	227375	44,6	282239	55,4	<0,001
Sim	62786	11,0	23474	37,4	39312	62,6	<0,001
Cardiopatia							
Não	119464	21,0	80152	67,1	39312	32,9	<0,001
Sim	449231	79,0	216109	48,1	233122	51,9	<0,001
Doença hematológica							
Não	441456	98,3	206166	46,7	235290	53,3	<0,001
Sim	7795	1,7	4180	53,6	3615	46,4	<0,001
Síndrome de Down							
Não	446779	99,6	209055	46,8	237724	53,2	<0,001
Sim	1594	0,4	755	47,4	839	52,6	0,04
Doença hepática							
Não	439323	98,1	204832	46,6	234491	53,4	<0,001
Sim	8686	1,9	5136	59,1	3550	40,9	<0,001
Asma							
Não	430936	95,3	202260	46,9	228676	53,1	<0,001
Sim	21168	4,7	8841	41,8	12327	58,2	<0,001
Diabetes							
Não	260000	45,6	120059	46,2	139941	53,8	<0,001
Sim	310429	54,4	150709	48,5	159720	51,5	<0,001
Doença neurológica							
Não	409022	87,7	187737	45,9	221285	54,1	<0,001
Sim	57534	12,3	32808	57,0	24726	43,0	<0,001
Pneumopatia							
Não	411704	88,5	189910	46,1	221794	53,9	<0,001
Sim	53559	11,5	29607	55,3	23952	44,7	<0,001
Imunodepressão							
Não	427735	94,5	198615	46,4	229120	53,6	<0,001
Sim	24879	5,5	13890	55,8	10989	44,2	<0,001
Doença renal							
Não	414401	89,7	189995	45,8	224406	54,2	<0,001
Sim	47703	10,3	28836	60,4	18867	39,6	<0,001
Obesidade							
Não	399785	87,4	185499	46,4	214286	53,6	<0,001
Sim	57889	12,6	29698	51,3	28191	48,7	<0,001
Vacina contra a COVID-19							
Não	145596	34,4	73067	50,2	72529	49,8	0,16
Sim	277117	65,6	110663	39,9	166454	60,1	<0,001
Admissão em UTI^||^							
Não	504578	57,8	149529	29,6	355049	70,4	<0,001
Sim	368945	42,2	247501	67,1	121444	32,9	<0,001

*SRAG = Síndrome Respiratória Aguda Grave; ^†^p-valor = Medida estatística que indica a probabilidade de que os resultados observados tenham ocorrido por acaso, assumindo que a hipótese nula (*H*_*0*_ ) seja verdadeira; ^‡^n = Tamanho da amostra; ^§^% = Porcentagem; ^||^UTI = Unidade de Terapia Intensiva

O percentual de óbitos por COVID-19 no sexo masculino (48,6%) foi menor quando comparado ao grupo que recebeu alta hospitalar (51,4%). O mesmo padrão foi observado entre pessoas idosas do sexo feminino, nas quais o percentual de óbitos por COVID-19 foi menor (44,4%) em comparação com aquelas que receberam alta hospitalar (55,6%). 

Quando analisada a escolaridade, o grupo com ensino superior apresentou menor percentual de óbito por COVID-19 (44,8%). Por outro lado, quando analisadas as pessoas idosas sem escolaridade, esse quadro se inverte, com maior percentual de óbitos por COVID-19 (57,1%). 

Em relação à zona de residência, observou-se maior percentual de óbitos por COVID-19 de pessoas idosas residentes em zonas periurbanas (51,8%). 

Quanto às características clínicas, as análises evidenciaram percentuais mais expressivos de óbitos por COVID-19 entre pessoas idosas com desconforto respiratório (51,4%), doença hematológica (53,6%), doença hepática (59,1%), doença neurológica (57%), pneumopatia (55,2%), imunodepressão (55,8%), doença renal (60,4%), obesidade (51,3%), admissão em UTI (67,1%) e uso de suporte ventilatório invasivo (85,4%).

A Tabela 2 mostra os resultados do teste de Kruskal-Wallis para comparação de medianas entre os grupos que evoluíram ou não a óbito por COVID-19, para as variáveis idade e dias de internação. O teste aponta diferenças significativas nas medianas para ambas as variáveis, mostrando que pessoas idosas que faleceram por COVID-19 apresentam maior mediana de idade e também maior tempo de internação do que aquelas que tiveram alta ou evoluíram a óbito por outras causas.

**Tabela 2:** Testes Kruskal-Wallis para comparação de medianas - Óbito por COVID-19 x Alta/Óbito por outras causas. Brasil, 2023

Variável	Evolução	Mediana	p-valor*
Idade	Alta/Óbito por outras causas	71	<0,001
	Óbito por COVID-19	74	
Dias de internação	Alta/Óbito por outras causas	7	<0,001
	Óbito por COVID-19	9	

*p-valor = Medida estatística que indica a probabilidade de que os resultados observados tenham ocorrido por acaso, assumindo que a hipótese nula (*H*_*0*_ ) seja verdadeira

A Figura 1 apresenta a linha temporal das taxas mensais de óbito entre as pessoas idosas deste estudo. A partir dela, é possível verificar que, logo após o início da vacinação, parece haver uma mudança de comportamento na taxa de mortalidade entre os indivíduos considerados.

**Figura 1:** Taxa de óbito em pessoas idosas por COVID-19 entre os casos registrados no período de março/2020 a março/2023

No entanto, para verificar as tendências dessa série temporal, bem como estimar pontos de inflexão (onde há mudança de comportamento), e comparar os comportamentos pré e pós-vacinal, foram utilizados testes de flutuação generalizados para estimar os *breakpoints*, o teste de Mann-Kendall para tendência, e o teste de Chow para comparação entre períodos da série.

Pelo algoritmo utilizado para estimar o ponto de mudança de tendência (*breakpoint*), verificou-se que a quantidade de quebras necessária para explicar a série, reduzindo os valores do Critério de Informação de Akaike (AIC) e da Soma dos Quadrados dos Resíduos (RSS), foi de 1 ponto de quebra, localizado em janeiro de 2021, coincidindo com o mês do início da vacinação.

A série foi dividida em duas segmentações, considerando como ponto de corte o início da vacinação (concomitantemente ao ponto de quebra estimado). O teste de Mann-Kendall estima que, antes do período vacinal, o valor de τ, referente à inclinação da série, era de -0,64, e após o início da vacinação o valor de τ passou a ser de -0,78, ambos significativos. Esse resultado mostra que, em ambos os períodos, a taxa de óbito entre pessoas idosas é decrescente; no entanto, passou a apresentar uma inclinação de decréscimo mais acentuada a partir do início da vacinação. O teste de Chow compara as séries em ambos os períodos e foi capaz de identificar diferenças significativas no comportamento da taxa de óbito antes e após o início da vacinação.

Nas análises univariadas, das 31 variáveis investigadas, cinco associaram-se ao desfecho óbito por COVID-19, as quais foram posteriormente incluídas no modelo de regressão ajustada (Tabela 3).

**Tabela 3:** Modelo ajustado das associações entre as características sociodemográficas e clínicas de pessoas idosas que evoluíram a óbito por COVID-19 no Brasil (n = 468.237). Brasil, 2023

Variáveis	OR*	IC^†^		p-valor^‡^
Sociodemográficas				
Faixa etária				
60 a 74 anos	1,00			
75 a 84 anos	1,77	1,74	1,79	<0,001
85 anos ou mais	3,01	2,96	3,06	<0,001
Raça/cor				
Amarela	1,00			
Branca	1,11	1,05	1,17	<0,001
Indígena	1,54	1,34	1,76	<0,001
Parda	1,28	1,22	1,35	<0,001
Preta	1,48	1,39	1,57	<0,001
Sexo				
Feminino	1,00			
Masculino	1,27	1,25	1,28	<0,001
Clínicas				
Vacina contra a COVID-19				
Não	1,00			
Sim	0,68	0,67	0,69	<0,001
Uso de suporte ventilatório				
Não	1,00			
Invasivo	19,54	19,14	19,95	<0,001
Não invasivo	1,78	1,75	1,80	<0,001

*OR = *Odds Ratio*; ^†^IC = Intervalo de Confiança; ^‡^p-valor = Medida estatística que indica a probabilidade de que os resultados observados tenham ocorrido por acaso, assumindo que a hipótese nula (*H*_*0*_ ) seja verdadeira

A faixa etária entre 75 e 84 anos associou-se ao óbito por COVID-19, com 77% maiores chances de morte por complicações da doença quando comparada a pessoas idosas com idade entre 60 e 74 anos. Nessa mesma comparação, no grupo com idade de 85 anos ou mais, as chances de evoluir para óbito por COVID-19 são três vezes maiores. 

No tocante à raça/cor, observou-se predominância de maiores chances de óbito por COVID-19 em pessoas idosas autodeclaradas indígenas (54%) e pretas (48%), quando comparadas à raça/cor amarela. 

Além disso, homens idosos apresentaram 1,27 vezes maiores chances de óbito por COVID-19 quando comparados a mulheres idosas. Pessoas idosas que receberam a vacina contra a COVID-19 reduziram em 32% as chances de óbito quando comparadas àquelas que não foram imunizadas. 

Em relação ao uso de suporte ventilatório, o uso do dispositivo associou-se ao óbito por COVID-19, com chances expressivas de morte entre pessoas idosas que utilizaram o sistema invasivo (19,54 vezes) e o não invasivo (1,78 vezes), quando comparadas aos hospitalizados que não fizeram uso desse recurso.

## Discussão

Neste estudo, o objetivo foi analisar os fatores associados ao óbito de pessoas idosas hospitalizadas por COVID-19 no Brasil. Os achados evidenciaram que as chances de a pessoa idosa evoluir para óbito por COVID-19 aumentam a depender de alguns fatores aos quais o indivíduo está condicionado. A idade avançada, somada a fatores clínicos, significa um prognóstico negativo e óbito pela COVID-19 nessa população^6^. 

Achados de um estudo conduzido no Irã com 1.694 pessoas idosas evidenciaram que a idade é um fator preditivo associado ao óbito por COVID-19^13^. Ter 75 anos de idade ou mais aumentou em mais de duas vezes as chances de óbito pela doença quando comparado à faixa etária de 60 a 74 anos. Além da idade, o estudo apontou que fatores como a unidade de hospitalização e indicadores laboratoriais foram os principais preditores do óbito por COVID-19^13^.

Estudos recentes destacam efeitos exacerbados e o óbito por COVID-19 em pessoas idosas associados à senescência celular, uma alteração funcional ligada ao envelhecimento, considerada um fator central na hiperinflamação desencadeada pelo SARS-CoV-2^14^ ^)-(^ ^16^. O vírus é capaz de induzir a senescência celular, intensificando o fenótipo secretor associado a essa condição, que consiste principalmente em moléculas pró-inflamatórias, enzimas degradadoras da matriz extracelular, ativadores do sistema complemento e substâncias pró-coagulantes liberadas por células senescentes^15^. Esses efeitos são especialmente agravados em pessoas idosas devido à maior presença de células senescentes em seus tecidos. Esse processo desencadeia tempestades de citocinas, infiltração de células imunes prejudiciais aos tecidos, inflamação endotelial, fibrose e formação de microtrombos, que contribuem para a falência de órgãos vitais e o óbito^14^.

É importante ressaltar que a associação da idade avançada com o desfecho óbito vai além das alterações celulares. Embora as análises deste estudo não tenham associado doenças pré-existentes ao óbito de pessoas idosas por COVID-19, a literatura aponta associações importantes entre comorbidades pré-existentes e aumento de risco de morte pela doença^7^. As chances de morte por COVID-19 aumentam em indivíduos com doenças cardiovasculares, renais, metabólicas, câncer e doenças respiratórias, como a Doença Pulmonar Obstrutiva Crônica (DPOC)^6^ ^,^ ^17^, considerada a terceira maior causa de morte no mundo, segundo ranking da Organização Mundial da Saúde, em 2021^18^. 

Outro fator importante associado a maiores chances de morte por COVID-19 é o sexo. Homens com 60 anos ou mais são mais propensos ao óbito pela doença quando comparados ao sexo feminino por diversos fatores. A idade avançada, somada a fatores ambientais e comportamentais, como o tabagismo, tornam os homens mais expostos. Além disso, a maior exposição do sexo masculino a complicações e óbito por COVID-19 pode ser atribuída a diferenças em hormônios sexuais associados a processos inflamatórios e à expressão reduzida da enzima conversora de angiotensina 2 (ECA2), que pode proteger os pulmões de lesões agudas decorrentes de lesões virais^19^. 

Em relação à raça/cor, achados de um estudo realizado com indígenas no estado do Amapá, no Brasil, apontaram que a idade avançada, somada a doenças cardiovasculares, aumenta em até quatro vezes as chances de óbito por COVID-19. Ainda, pessoas idosas residentes na região metropolitana tiveram as chances de óbito por COVID-19 aumentadas em 2,90 vezes quando comparadas aos indígenas residentes em regiões do interior do estado^20^. 

Pessoas idosas que se autodeclaram negras enfrentam maior vulnerabilidade à infecção e ao óbito por COVID-19 em decorrência de desafios sociais, como baixos rendimentos, barreiras no acesso a serviços de saúde, desconhecimento sobre a enfermidade, propagação de informações equivocadas, ausência de políticas públicas culturalmente sensíveis, discriminação racial no âmbito da saúde e hesitação quanto à eficácia das vacinas contra a COVID-19^21^. Ademais, estudos indicam que a combinação de idade avançada e pertencimento à raça/cor negra está fortemente associada a um risco elevado de hospitalizações, admissões em unidades de terapia intensiva, complicações clínicas e óbitos pela doença^22^.

No tocante à vacina contra a COVID-19, achados de um estudo de meta-análise apontam um fator de proteção em pessoas idosas que receberam o imunizante. Em uma análise com mais de 3 milhões de participantes, pessoas idosas imunizadas apresentaram redução nas chances de infecção e óbito por COVID-19 em mais de 80%^23^. Ainda, estudos recentes têm reforçado que a proteção conferida pela vacinação nesse grupo deve ser interpretada à luz de dois fenômenos complementares: a imunossenescência e o declínio temporal da efetividade vacinal, sobretudo para desfechos de infecção e doença sintomática, com preservação relativamente maior para eventos graves. Nesse sentido, uma meta-análise internacional evidenciou que a efetividade tende a reduzir progressivamente com o passar dos meses e que doses de reforço restabelecem níveis mais elevados de proteção, elemento particularmente relevante para pessoas idosas, nas quais a resposta imune costuma ser menos robusta e mais heterogênea^24^. 

Em consonância, análises em redes assistenciais e de vigilância de efetividade em contextos reais têm mostrado benefício adicional de vacinas atualizadas contra variantes recentes, com impacto mais nítido em faixas etárias avançadas. Estimativas de efetividade em adultos mais velhos apontam redução importante de hospitalização após vacinação sazonal recente, reforçando a necessidade de estratégias de reforço direcionadas a pessoas idosas e grupos vulneráveis, inclusive em cenários de predominância de sublinhagens Ômicron geneticamente divergentes de composições vacinais anteriores^25^ ^)-(^ ^27^. 

Quanto ao uso de suporte ventilatório, pessoas idosas hospitalizadas por COVID-19 admitidas em UTI têm as chances de óbito pela doença aumentadas quando utilizam esse recurso. Um estudo realizado com 1.994 participantes revelou que indivíduos que utilizaram suporte ventilatório não invasivo e invasivo apresentaram aumento de 2,25 e 24 vezes, respectivamente, nas chances de óbito por COVID-19 quando comparados àqueles que não necessitaram do dispositivo^28^. 

Também é importante destacar que, além da gravidade intrínseca do quadro, a trajetória de escalonamento do suporte ventilatório pode influenciar os desfechos. Em pacientes com insuficiência respiratória aguda por COVID-19, o uso de estratégias não invasivas (como cânula nasal de alto fluxo e ventilação não invasiva) tem sido associado a benefícios em subgrupos selecionados; contudo, falhas nessas modalidades, quando acompanhadas de retardo na intubação, podem estar relacionadas a pior prognóstico^29^. Estudos multicêntricos demonstraram maior mortalidade hospitalar em pacientes submetidos à intubação tardia em comparação à intubação precoce, reforçando a importância de monitorização rigorosa, critérios objetivos de falha e decisão clínica oportuna, sobretudo em pessoas idosas, nas quais a reserva fisiológica é menor^30^. 

Além disso, a ventilação mecânica invasiva nesse grupo em específico associa-se com maior frequência a desfechos adversos relacionados à internação em UTI, como delirium, fraqueza adquirida, prolongamento de sedação e complicações infecciosas, particularmente pneumonia associada à ventilação mecânica, com impacto sobre o tempo de permanência e a mortalidade^31^ ^)-(^ ^32^. Estudos com pessoas idosas em faixas etárias mais avançadas também apontam piores resultados quando a ventilação não invasiva é requerida em cenários pandêmicos, sugerindo que idade avançada, fragilidade e gravidade respiratória tendem a se somar como determinantes de pior evolução clínica^33^. 

No presente estudo, destacam-se algumas limitações inerentes ao uso de dados secundários oriundos de sistemas administrativos de saúde, os quais podem conter subnotificações ou informações incompletas em determinadas variáveis. Contudo, a metodologia analítica adotada foi criteriosamente escolhida para minimizar o impacto de dados ausentes sobre as análises realizadas, assegurando maior rigor e confiabilidade nas inferências relacionadas à saúde. Os resultados estatísticos que não associaram comorbidades pré-existentes ao óbito por COVID-19 neste estudo podem estar relacionados ao fato de não haver um método de análise padronizado para pesquisas dessa natureza.

Apesar dessas restrições, os resultados alcançados possibilitaram uma análise aprofundada das características epidemiológicas e clínicas da infecção e do óbito de pessoas idosas por COVID-19 no Brasil. Além disso, reforça-se o papel de estudos com esse delineamento metodológico como propulsores de investigações similares em outras localidades, ampliando a compreensão sobre os determinantes contextuais e o comportamento da doença em diferentes estratos populacionais. Essa abordagem destaca a relevância da produção científica voltada para a aplicação prática e para a geração de benefícios concretos à sociedade.

Nesse sentido, os achados deste estudo configuram-se como subsídio estratégico para gestores de saúde, oferecendo embasamento científico para a formulação, qualificação e implementação de políticas públicas que reflitam a realidade vivenciada pela população idosa. Ao considerar as vulnerabilidades existentes, tais políticas podem promover intervenções mais resolutivas e eficazes, garantindo impacto positivo nas ações de saúde e fortalecendo a equidade e a qualidade da assistência prestada.

## Conclusão

Os fatores associados ao óbito por COVID-19 de pessoas idosas hospitalizadas estiveram relacionados à idade, sexo, raça/cor, vacina contra a COVID-19 e uso de suporte ventilatório. Além disso, ter 80 anos ou mais, ser do sexo masculino, ser de etnia indígena e ter feito uso de suporte ventilatório invasivo foram condições associadas às chances mais expressivas de óbito.

A administração da vacina contra a COVID-19 demonstrou-se eficaz ao proporcionar proteção às pessoas idosas expostas ao SARS-CoV-2, evidenciando a importância das estratégias preventivas, como a imunização, na minimização dos impactos adversos da pandemia sobre um grupo populacional mais suscetível. Essa relação enfatiza a necessidade de manter e intensificar as campanhas de vacinação como estratégia de saúde coletiva, a fim de minimizar as repercussões multissistêmicas da COVID-19 para a vida, a saúde e o bem-estar das pessoas idosas.

Destaca-se a importância do avanço das estratégias de monitoramento da saúde da pessoa idosa, especialmente direcionadas à ampliação da imunoprevenção e à diminuição da desinformação que permeia as vacinas contra a COVID-19. Almeja-se que o presente estudo contribua como base para o desenvolvimento de iniciativas que atendam de forma plena às demandas específicas da população idosa. Entre essas iniciativas, ressalta-se o fortalecimento das ações de saúde no nível da atenção básica, visando ao acesso oportuno aos serviços de saúde e ao cuidado continuado, permeado pelas necessidades individuais das pessoas idosas, buscando mitigar as vulnerabilidades dessa faixa etária frente à COVID-19.

## Data Availability

Todos os dados gerados ou analisados durante este estudo estão incluídos neste artigo publicado.

# Translation: Factores asociados a la muerte de personas mayores hospitalizadas por COVID-19 en Brasil*

**DOI:** 10.1590/1518-8345.8031.4944

**Authors:** Luiz Hiroshi Inoue, Wanessa Cristina Baccon, Francielle Renata Danielli Martins Marques, Maria Aparecida Salci, Vanessa Denardi Antoniassi Baldissera, Stéfane Lele Rossoni, Lígia Carreira

**Affiliations:** 1 Universidade Estadual de Maringá́, Maringá, PR, Brasil.

**Keywords:** Anciano, Mortalidad, Hospitalización, COVID-19, Enfermería, Factores de Riesgo

## Abstract

**Objetivo:**

analizar los factores asociados a la muerte de personas mayores hospitalizadas por COVID-19 en Brasil.

**Método:**

estudio transversal realizado con datos secundarios de dominio público de personas mayores hospitalizadas que evolucionaron a muerte por COVID-19. Las razones de probabilidades se obtuvieron mediante el ajuste de modelo de regresión logística.

**Resultados:**

el análisis estuvo compuesto por 468.239 muertes de personas mayores hospitalizadas en Brasil. Algunos factores se asociaron con la muerte, tales como: edad entre 75 y 84 años (OR=1,77; IC_95%_=1,74-1,79) y 85 años o más (OR=3,01; IC_95%_=2,96-3,06); raza/color blanca (OR=1,11; IC_95%_=1,05-1,17), indígena (OR=1,54; IC_95%_=1,34-1,76), parda (OR=1,28; IC_95%_=1,22-1,35) y negra (OR=1,48; IC_95%_=1,39-1,57); sexo masculino (OR=1,27; IC_95%_=1,25-1,28); vacuna contra la COVID-19 (OR=0,68; IC_95%_=0,67-0,69); uso de soporte ventilatorio invasivo (OR=19,54; IC_95%_=19,14-19,95) y no invasivo (OR=1,78; IC_95%_=1,75-1,80).

**Conclusión:**

se identificó asociación entre edad, sexo, raza/color, vacunación contra la COVID-19 y uso de soporte ventilatorio con la muerte por COVID-19.

## Destacados

**(1)** Factores asociados a la muerte por COVID-19 en personas mayores hospitalizadas.

**(2)** Condiciones asociadas a mayores probabilidades de muerte por COVID-19 en personas mayores.

**(3)** Eficacia de la vacuna contra la COVID-19 en personas mayores como estrategia preventiva.

## Introducción

La pandemia de *coronavirus disease* 2019 (COVID-19) causó miles de muertes a nivel mundial, y la mortalidad en poblaciones de riesgo continúa siendo ampliamente discutida en la literatura como una forma de promover medidas de prevención y mejoras en la salud para estos grupos^1^. Debido a su alta capacidad de transmisión, en menos de seis meses de pandemia la enfermedad ya había cobrado más de un millón de vidas^2^, acumulando más de siete millones de muertes en todo el mundo^3^. En Brasil, datos de febrero de 2025 indican que el número de víctimas fatales por COVID-19 alcanza casi 715 mil muertes^4^. 

La población de personas mayores fue la más afectada en términos de mortalidad por COVID-19, presentando mayores probabilidades de muerte en comparación con personas más jóvenes^5^. La edad avanzada y el diagnóstico de enfermedades preexistentes son factores asociados con peores desenlaces de la COVID-19^6^. 

La literatura identifica importantes predictores de complicaciones de la enfermedad, tales como marcadores genéticos y ambientales, inmunosupresión, enfermedades cardiovasculares, respiratorias y gastrointestinales, lugar de residencia y admisión en Unidad de Cuidados Intensivos (UCI)^7^. De este modo, el grupo de personas mayores se clasifica como un grupo de alto riesgo, debido a factores relacionados con el proceso de envejecimiento, como alteraciones fisiológicas y múltiples comorbilidades, lo que hace que esta población sea más susceptible a contraer la enfermedad y evolucionar hacia la muerte^8^. 

El análisis del impacto de la COVID-19 en la población de personas mayores permite identificar factores asociados a la mortalidad, así como comprender las manifestaciones clínicas que pueden comprometer la calidad de vida y ocasionar agravios a la salud a corto y largo plazo^9^. Esta investigación no solamente contribuye al avance del conocimiento científico sobre la enfermedad, sino que también ofrece insumos esenciales para la formulación de estrategias de salud pública dirigidas a este grupo etario.

Al comprender los determinantes que influyen en el desenlace clínico de la COVID-19 en personas mayores, los gestores de salud pueden mejorar las políticas públicas que prioricen el monitoreo continuo de esta población, reconocida como particularmente vulnerable durante la pandemia. En este sentido, dada la vulnerabilidad intrínseca de la población de personas mayores a desenlaces graves de la enfermedad, se formuló la hipótesis de que variables sociodemográficas y clínicas pueden estar asociadas con la muerte de personas mayores por COVID-19. De esta forma, el objetivo del presente estudio fue analizar los factores asociados a la muerte de personas mayores hospitalizadas por COVID-19 en Brasil.

## Método

### Diseño del estudio

Se trata de un estudio transversal delineado con base en datos secundarios de acceso público, que aborda la muerte de personas mayores hospitalizadas como consecuencia de la COVID-19. La conducción de la investigación siguió los criterios establecidos por la herramienta *Strengthening the Reporting of Observational Studies in Epidemiology* (STROBE), garantizando rigor metodológico y transparencia en la presentación de los hallazgos^10^.

El presente estudio está vinculado a una investigación multicéntrica, realizada en colaboración entre la *Universidade Estadual de Maringá* (UEM) y la Secretaría de Salud del Estado de Paraná (SESA/PR), con financiamiento del *Conselho Nacional de Desenvolvimento Científico e Tecnológico* (CNPq).

### Lugar

La investigación se llevó a cabo a nivel nacional, abarcando todo el territorio brasileño. De acuerdo con estimaciones del *Instituto Brasileiro de Geografia e Estatística* (IBGE) para el año 2022, la población del país está compuesta por aproximadamente 203 millones de personas, de las cuales 32.113.490 pertenecen al grupo etario de personas mayores^11^.

### Población

En el período analizado se observaron 1.070.336 casos de personas mayores diagnosticadas con Síndrome Respiratorio Agudo Grave (SRAG), residentes en todo el territorio brasileño, entre marzo de 2020 y marzo de 2023. 

### Definición de la muestra

Se seleccionaron individuos considerados personas mayores, es decir, aquellos con edad igual o superior a 60 años, conforme a lo dispuesto en el artículo 1º del Estatuto del Adulto Mayor (Ley 10.741/2003) - Brasil, que desarrollaron SRAG y fueron hospitalizados por COVID-19.

### Variables del estudio

La variable dependiente se refiere al desenlace del paciente con relación a la notificación, clasificada en muerte por COVID-19 o alta hospitalaria/muerte por otras causas. Su determinación se basó en el análisis del campo específico indicado en la ficha de notificación correspondiente.

Las variables independientes se distribuyeron en dos grupos principales: variables sociodemográficas y variables clínicas. Para las características sociodemográficas se consideraron: grupo etario (60 a 74; 75 a 84; 85 años o más), sexo (femenino; masculino), raza/color (blanca; negra; amarilla; indígena; parda), escolaridad (Educación Primaria I; Educación Primaria II; Educación Secundaria; sin escolaridad; Educación Superior) y zona de residencia (periurbana; rural; urbana).

Las variables clínicas incluyeron síntomas como: fiebre (sí; no), tos (sí; no), dolor de garganta (sí; no), disnea (sí; no), malestar respiratorio (sí; no), saturación < 95% (sí; no), diarrea (sí; no), vómito (sí; no), dolor abdominal (sí; no), fatiga (sí; no), pérdida del olfato (sí; no) y pérdida del gusto (sí; no). También se incluyeron comorbilidades como: cardiopatía (sí; no), enfermedad hematológica (sí; no), síndrome de Down (sí; no), enfermedad hepática (sí; no), asma (sí; no), diabetes (sí; no), enfermedad neurológica (sí; no), neumopatía (sí; no), inmunodepresión (sí; no), enfermedad renal (sí; no) y obesidad (sí; no). Además, se consideraron otros factores, como: vacuna contra la COVID-19 (sí; no), admisión en UCI (sí; no) y uso de soporte ventilatorio (no; invasivo; no invasivo).

### Recolección de datos

La información fue extraída de fichas de registro individual de casos de SRAG disponibles en la plataforma SIVEP-Gripe, instrumento del Sistema de Vigilancia Epidemiológica vinculado a la Secretaría de Vigilancia en Salud (SVS) para el monitoreo de casos y muertes por SRAG^12^. El período analizado comprendió desde marzo de 2020 hasta marzo de 2023, seleccionado con el propósito de abarcar los datos más actualizados en el momento de la recolección.

### Tratamiento y análisis de los datos

Los datos fueron organizados en hojas de cálculo electrónicas y procesados en el programa estadístico R Core Team (2024), versión 4.4.2. Para el análisis descriptivo de los datos se calcularon las frecuencias absolutas y relativas de las variables categóricas con relación al desenlace, utilizándose la prueba de chi-cuadrado de Pearson para establecer comparaciones. Para las variables numéricas se calcularon las medidas resumen (mínimo, media, máximo y desviación estándar).

Las pruebas de comparación realizadas para verificar diferencias significativas entre los grupos que evolucionaron a muerte o no fueron: prueba t de Student, Mann-Whitney, Kruskal-Wallis y ANOVA. 

El análisis de los factores asociados al desenlace de muerte por COVID-19 se realizó mediante un modelo de regresión logística de la familia binomial. Se consideró 1 para la muerte de la persona mayor por COVID-19 y 0 para alta o muerte por otras causas. El nivel de significancia considerado en este análisis fue del 5% y el intervalo de confianza del 95%.

### Aspectos éticos

Al tratarse de un estudio realizado con datos secundarios de dominio público, se dispensó la evaluación por parte de un Comité de Ética en Investigación. Sin embargo, el estudio multicéntrico al cual está vinculada la presente investigación obtuvo dictamen favorable del Comité de Ética en Investigación de la *Universidade Estadual de Maringá*, bajo el dictamen nº 4.165.272 y CAAE: 34787020.0.0000.0104. Asimismo, recibió aprobación de la Secretaría de Salud del Estado de Paraná, representada por el *Hospital do Trabalhador*, obteniendo el dictamen nº 4.214.589 y CAAE: 34787020.0.3001.5225, el 15 de agosto de 2020. 

## Resultados

Se analizaron 1.064.033 registros de personas mayores hospitalizadas con diagnóstico confirmado de SRAG por COVID-19, de los cuales 468.237 casos evolucionaron a muerte por la enfermedad y 536.997 recibieron alta hospitalaria. La edad mínima, media y máxima observada entre los casos confirmados de COVID-19 que evolucionaron a muerte fue de 60, 75,3 y 104 años, respectivamente, con una desviación estándar de 9,55.

Se observó un aumento gradual en el porcentaje de muertes conforme al avance de la edad. Aunque el porcentaje de muertes por COVID-19 en el grupo etario de 60 a 74 años (41%) fue menor en comparación con quienes recibieron alta hospitalaria (58,9%), este patrón se invierte en los grupos etarios de 75 a 84 años (51%) y de 85 años o más (58,2%), en comparación con los grupos de la misma edad que recibieron alta hospitalaria. Con relación a la raza/color, se observó una mayor proporción de muertes por COVID-19 entre personas mayores autodeclaradas negras (53,1%) e indígenas (52,3%) (Tabla 1). 

**Tabla 1:** Características sociodemográficas y clínicas de personas mayores hospitalizadas por SRAG* que evolucionaron a muerte por COVID-19 en Brasil (n = 468.237). Brasil, 2023

Variables	Evolución del caso						Valor de p^†^
	Hospitalizados		Muertes		Alta hospitalaria		
	(n = 1.064.033)		(n = 468.237)		(n = 536.997)		
	n^‡^	%^§^	n^‡^	%^§^	n^‡^	%^§^	
Sociodemográficas							
Grupo etario							
60 a 74 años	570869	56,8	234386	41,1	336483	58,9	<0,001
75 a 84 años	278597	27,7	143150	51,4	135447	48,6	<0,001
85 años o más	155768	15,5	90701	58,2	65067	41,8	<0,001
Raza/Color							
Amarilla	10568	1,3	4714	44,6	5854	55,4	<0,001
Blanca	456824	55,1	211448	46,3	245376	53,7	<0,001
Indígena	1766	0,2	923	52,3	843	47,7	0,06
Parda	317314	38,3	156533	49,3	160781	50,7	<0,001
Negra	42909	5,2	22800	53,1	20109	46,9	<0,001
Sexo							
Femenino	482487	48,0	214093	44,4	268394	55,6	<0,001
Masculino	522646	52,0	254099	48,6	268547	51,4	<0,001
Escolaridad							
Educación Primaria I	136204	39,2	70381	51,7	65823	48,3	<0,001
Educación Primaria II	66333	19,1	33342	50,3	32991	49,7	0,17
Educación Secundaria	70897	20,4	34385	48,5	36512	51,5	<0,001
Sin escolaridad	38603	11,1	22067	57,2	16536	42,8	<0,001
Educación Superior	35239	10,1	15774	44,8	19465	55,2	<0,001
Zona de Residencia							
Periurbana	3043	0,3	1577	51,8	1466	48,2	0,04
Rural	47792	5,4	22764	47,6	25028	52,4	<0,001
Urbana	837953	94,3	388237	46,3	449716	53,7	<0,001
Clínicas							
Fiebre							
No	309459	38,1	140075	45,3	169384	54,7	<0,001
Sí	502416	61,9	229594	45,7	272822	54,3	<0,001
Tos							
No	199616	23,3	98491	49,3	101125	50,7	<0,001
Sí	656076	76,7	287337	43,8	368739	56,2	<0,001
Dolor de garganta							
No	538834	78,8	245546	45,6	293288	54,4	<0,001
Sí	145360	21,2	61507	42,3	83853	57,7	<0,001
Disnea							
No	169020	19,4	58041	34,3	110979	65,7	<0,001
Sí	701491	80,6	349247	49,8	352244	50,2	<0,001
Malestar respiratorio							
No	242232	30,2	87892	36,3	154340	63,7	<0,001
Sí	560223	69,8	288193	51,4	272030	48,6	<0,001
Saturación < 95%							
No	185602	22,1	65109	35,1	120493	64,9	<0,001
Sí	654810	77,9	327136	50,0	327674	50,0	<0,001
Diarrea							
No	563198	83,2	256225	45,5	306973	54,5	<0,001
Sí	113625	16,8	47791	42,1	65834	57,9	<0,001
Vómito							
No	592319	89,2	268438	45,3	323881	54,7	<0,001
Sí	71840	10,8	30121	41,9	41719	58,1	<0,001
Dolor abdominal							
No	525005	91,9	232288	44,2	292717	55,8	<0,001
Sí	46073	8,1	19356	42,0	26717	58,0	<0,001
Fatiga							
No	397366	65,2	175839	44,3	221527	55,7	<0,001
Sí	212032	34,8	92916	43,8	119116	56,2	<0,001
Pérdida del olfato							
No	512583	89,5	228190	44,5	284393	55,5	<0,001
Sí	60133	10,5	22956	38,2	37177	61,8	<0,001
Pérdida del gusto							
No	509614	89,0	227375	44,6	282239	55,4	<0,001
Sí	62786	11,0	23474	37,4	39312	62,6	<0,001
Cardiopatía							
No	119464	21,0	80152	67,1	39312	32,9	<0,001
Sí	449231	79,0	216109	48,1	233122	51,9	<0,001
Enfermedad hematológica							
No	441456	98,3	206166	46,7	235290	53,3	<0,001
Sí	7795	1,7	4180	53,6	3615	46,4	<0,001
Síndrome de Down							
No	446779	99,6	209055	46,8	237724	53,2	<0,001
Sí	1594	0,4	755	47,4	839	52,6	0,04
Enfermedad hepática							
No	439323	98,1	204832	46,6	234491	53,4	<0,001
Sí	8686	1,9	5136	59,1	3550	40,9	<0,001
Asma							
No	430936	95,3	202260	46,9	228676	53,1	<0,001
Sí	21168	4,7	8841	41,8	12327	58,2	<0,001
Diabetes							
No	260000	45,6	120059	46,2	139941	53,8	<0,001
Sí	310429	54,4	150709	48,5	159720	51,5	<0,001
Enfermedad neurológica							
No	409022	87,7	187737	45,9	221285	54,1	<0,001
Sí	57534	12,3	32808	57,0	24726	43,0	<0,001
Neumopatía							
No	411704	88,5	189910	46,1	221794	53,9	<0,001
Sí	53559	11,5	29607	55,3	23952	44,7	<0,001
Inmunodepresión							
No	427735	94,5	198615	46,4	229120	53,6	<0,001
Sí	24879	5,5	13890	55,8	10989	44,2	<0,001
Enfermedad renal							
No	414401	89,7	189995	45,8	224406	54,2	<0,001
Sí	47703	10,3	28836	60,4	18867	39,6	<0,001
Obesidad							
No	399785	87,4	185499	46,4	214286	53,6	<0,001
Sí	57889	12,6	29698	51,3	28191	48,7	<0,001
Vacuna contra la COVID-19							
No	145596	34,4	73067	50,2	72529	49,8	0,16
Sí	277117	65,6	110663	39,9	166454	60,1	<0,001
Admisión en UCI^||^							
No	504578	57,8	149529	29,6	355049	70,4	<0,001
Sí	368945	42,2	247501	67,1	121444	32,9	<0,001

*SRAG = S*í*ndrome Respiratorio Agudo Grave; ^†^Valor de p = Medida estadística que indica la probabilidad de que los resultados observados hayan ocurrido por azar, asumiendo que la hipótesis nula (*H*_*0*_ ) sea verdadera; ^‡^n = Tamaño de la muestra; ^§^% = Porcentaje; ^||^UCI = Unidad de Cuidados Intensivos

El porcentaje de muertes por COVID-19 en el sexo masculino (48,6%) fue menor en comparación con el grupo que recibió alta hospitalaria (51,4%). El mismo patrón se observó entre las personas mayores del sexo femenino, en las cuales el porcentaje de muertes por COVID-19 fue menor (44,4%) en comparación con aquellas que recibieron alta hospitalaria (55,6%). 

Al analizar la escolaridad, el grupo con educación superior presentó el menor porcentaje de muerte por COVID-19 (44,8%). Por el contrario, entre las personas mayores sin escolaridad se observó un mayor porcentaje de muertes por COVID-19 (57,1%). 

Con relación a la zona de residencia, se observó un mayor porcentaje de muertes por COVID-19 entre personas mayores residentes en zonas periurbanas (51,8%). 

En cuanto a las características clínicas, los análisis evidenciaron porcentajes más elevados de muertes por COVID-19 entre personas mayores con malestar respiratorio (51,4%), enfermedad hematológica (53,6%), enfermedad hepática (59,1%), enfermedad neurológica (57%), neumopatía (55,2%), inmunodepresión (55,8%), enfermedad renal (60,4%), obesidad (51,3%), admisión en UCI (67,1%) y uso de soporte ventilatorio invasivo (85,4%).

La Tabla 2 muestra los resultados de la prueba de Kruskal-Wallis para la comparación de medianas entre los grupos que evolucionaron o no a muerte por COVID-19, para las variables edad y días de hospitalización. La prueba indica diferencias significativas en las medianas para ambas variables, mostrando que las personas mayores que fallecieron por COVID-19 presentan una mayor mediana de edad y también un mayor tiempo de hospitalización que aquellas que recibieron el alta o que fallecieron por otras causas.

**Tabla 2:** Prueba de Kruskal-Wallis para la comparación de medianas - Muerte por COVID-19 x Alta/Muerte por otras causas. Brasil, 2023

Variable	Evolución	Mediana	Valor de p*
Edad	Alta/Muerte por otras causas	71	<0,001
	Muerte por COVID-19	74	
Días de hospitalización	Alta/Muerte por otras causas	7	<0,001
	Muerte por COVID-19	9	

*Valor de p = medida estadística que indica la probabilidad de que los resultados observados hayan ocurrido por azar, asumiendo que la hipótesis nula (*H*_*0*_ ) sea verdadera

La Figura 1 presenta la línea temporal de las tasas mensuales de muerte entre las personas mayores incluidas en este estudio. A partir de ella, es posible observar que, poco después del inicio de la vacunación, parece haber un cambio en el comportamiento de la tasa de mortalidad entre los individuos analizados.

**Figura 1:** Tasa de muerte por COVID-19 en personas mayores entre los casos registrados en el período de marzo/2020 a marzo/2023

Sin embargo, para verificar las tendencias de esta serie temporal, así como estimar puntos de inflexión (donde ocurre un cambio en el comportamiento), y comparar los comportamientos en los períodos previo y posterior a la vacunación, se utilizaron pruebas de fluctuación generalizadas para estimar los *breakpoints*, la prueba de Mann-Kendall para analizar la tendencia, y la prueba de Chow para comparar los períodos de la serie.

Según el algoritmo utilizado para estimar el punto de cambio de tendencia (*breakpoint*), se verificó que el número de quiebres necesario para explicar la serie, reduciendo los valores del Criterio de Información de Akaike (AIC) y de la Suma de los Cuadrados de los Residuos (RSS), fue de un punto de quiebre, localizado en enero de 2021, coincidiendo con el mes de inicio de la vacunación.

La serie fue dividida en dos segmentos, considerando como punto de corte el inicio de la vacunación (coincidiendo con el punto de quiebre estimado). La prueba de Mann-Kendall estima que, antes del período de vacunación, el valor de τ, correspondiente a la inclinación de la serie, era de -0,64, y después del inicio de la vacunación el valor de τ pasó a ser de -0,78, ambos significativos. Este resultado muestra que, en ambos períodos, la tasa de muerte entre las personas mayores presenta una tendencia decreciente; sin embargo, el descenso se volvió más pronunciado a partir del inicio de la vacunación. La prueba de Chow compara las series en ambos períodos y permitió identificar diferencias significativas en el comportamiento de la tasa de muerte antes y después del inicio de la vacunación.

En los análisis univariados, de las 31 variables investigadas, cinco se asociaron con el desenlace de muerte por COVID-19, las cuales posteriormente fueron incluidas en el modelo de regresión ajustado (Tabla 3).

**Tabla 3:** Modelo ajustado de las asociaciones entre las características sociodemográficas y clínicas de personas mayores que evolucionaron a muerte por COVID-19 en Brasil (n = 468.237). Brasil, 2023

Variables	OR*	IC^†^		Valor de p^‡^
Sociodemográficas				
Grupo etario				
60 a 74 años	1,00			
75 a 84 años	1,77	1,74	1,79	<0,001
85 años o más	3,01	2,96	3,06	<0,001
Raza/color				
Amarilla	1,00			
Blanca	1,11	1,05	1,17	<0,001
Indígena	1,54	1,34	1,76	<0,001
Parda	1,28	1,22	1,35	<0,001
Negra	1,48	1,39	1,57	<0,001
Sexo				
Femenino	1,00			
Masculino	1,27	1,25	1,28	<0,001
Clínicas				
Vacuna contra la COVID-19				
No	1,00			
Sí	0,68	0,67	0,69	<0,001
Uso de soporte ventilatorio				
No	1,00			
Invasivo	19,54	19,14	19,95	<0,001
No invasivo	1,78	1,75	1,80	<0,001

*OR = *Odds Ratio*; ^†^IC = Intervalo de Confianza; ^‡^Valor de p = medida estadística que indica la probabilidad de que los resultados observados hayan ocurrido por azar, asumiendo que la hipótesis nula (*H*_*0*_ ) sea verdadera

El grupo etario de 75 a 84 años se asoció con la muerte por COVID-19, con un 77% más de probabilidades de muerte por complicaciones de la enfermedad en comparación con las personas mayores de 60 a 74 años. En esta misma comparación, en el grupo de 85 años o más, las probabilidades de evolucionar a muerte por COVID-19 fueron tres veces mayores. 

En cuanto a la raza/color, se observó predominio de mayores probabilidades de muerte por COVID-19 en personas mayores autodeclaradas indígenas (54%) y negras (48%), en comparación con la raza/color amarilla. 

Además, los hombres mayores presentaron 1,27 veces más probabilidades de muerte por COVID-19 en comparación con las mujeres mayores. Las personas mayores que recibieron la vacuna contra la COVID-19 redujeron en 32% las probabilidades de muerte en comparación con aquellas que no fueron inmunizadas. 

Con relación al uso de soporte ventilatorio, el uso de este recurso se asoció con la muerte por COVID-19, con probabilidades expresivas de muerte entre las personas mayores que utilizaron el sistema invasivo (19,54 veces) y no invasivo (1,78 veces), en comparación con los pacientes hospitalizados que no utilizaron este recurso.

## Discusión

En este estudio, el objetivo fue analizar los factores asociados a la muerte de personas mayores hospitalizadas por COVID-19 en Brasil. Los hallazgos evidenciaron que las probabilidades de que una persona mayor evolucione hacia la muerte por COVID-19 aumentan dependiendo de algunos factores a los que el individuo está expuesto. La edad avanzada, sumada a factores clínicos, se asocia con un pronóstico desfavorable y muerte por COVID-19 en esta población^6^. 

Los hallazgos de un estudio realizado en Irán con 1.694 personas mayores evidenciaron que la edad es un factor predictivo asociado a la muerte por COVID-19^13^. Tener 75 años o más incrementó en más de dos veces las probabilidades de muerte por la enfermedad en comparación con el grupo etario de 60 a 74 años. Además de la edad, el estudio señaló que factores como la unidad de hospitalización e indicadores de laboratorio fueron los principales predictores de muerte por COVID-19^13^.

Estudios recientes destacan efectos exacerbados y la muerte por COVID-19 en personas mayores asociados a la senescencia celular, una alteración funcional vinculada al envejecimiento, considerada un factor central en la hiperinflamación desencadenada por el SARS-CoV-2^14^ ^)-(^ ^16^. El virus es capaz de inducir la senescencia celular, intensificando el fenotipo secretor asociado a esta condición, que consiste principalmente en moléculas proinflamatorias, enzimas degradadoras de la matriz extracelular, activadores del sistema del complemento y sustancias procoagulantes liberadas por células senescentes^15^. Estos efectos se agravan especialmente en las personas mayores debido a la mayor presencia de células senescentes en sus tejidos. Este proceso desencadena tormentas de citocinas, infiltración de células inmunes perjudiciales para los tejidos, inflamación endotelial, fibrosis y formación de microtrombos, lo que contribuye a la falla de órganos vitales y a la muerte^14^.

Es importante señalar que la asociación entre la edad avanzada y el desenlace de muerte va más allá de las alteraciones celulares. Aunque los análisis de este estudio no asociaron enfermedades preexistentes con la muerte de personas mayores por COVID-19, la literatura reporta importantes asociaciones entre comorbilidades preexistentes y el aumento del riesgo de muerte por la enfermedad^7^. Las probabilidades de muerte por COVID-19 aumentan en individuos con enfermedades cardiovasculares, renales, metabólicas, cáncer y enfermedades respiratorias, como la Enfermedad Pulmonar Obstructiva Crónica (EPOC)^6^ ^),(^ ^17^, considerada la tercera causa de muerte en el mundo, según el ranking de la Organización Mundial de la Salud, en 2021^18^. 

Otro factor importante asociado a mayores probabilidades de muerte por COVID-19 es el sexo. Los hombres de 60 años o más son más propensos a morir por la enfermedad en comparación con las mujeres por diversos factores. La edad avanzada, sumada a factores ambientales y conductuales, como el tabaquismo, hace que los hombres estén más expuestos. Además, la mayor exposición del sexo masculino a complicaciones y muerte por COVID-19 puede atribuirse a diferencias en hormonas sexuales asociadas a procesos inflamatorios y a la expresión reducida de la enzima convertidora de angiotensina 2 (ECA2), que puede proteger los pulmones frente a lesiones agudas derivadas de infecciones virales^19^. 

Con relación a la raza/color, los hallazgos de un estudio realizado con poblaciones indígenas en el estado de Amapá, en Brasil, señalaron que la edad avanzada, sumada a enfermedades cardiovasculares, aumenta hasta cuatro veces las probabilidades de muerte por COVID-19. Asimismo, las personas mayores residentes en la región metropolitana presentaron probabilidades de muerte por COVID-19 2,90 veces mayores en comparación con los indígenas residentes en regiones del interior del estado^20^. 

Las personas mayores que se autodeclaran negras enfrentan mayor vulnerabilidad a la infección y a la muerte por COVID-19 debido a desafíos sociales, como bajos ingresos, barreras en el acceso a servicios de salud, desconocimiento sobre la enfermedad, propagación de información errónea, ausencia de políticas públicas culturalmente sensibles, discriminación racial en el ámbito sanitario y dudas respecto a la eficacia de las vacunas contra la COVID-19^21^. Además, estudios indican que la combinación de edad avanzada y pertenencia a la raza/color negra está fuertemente asociada con un mayor riesgo de hospitalización, admisión en unidades de cuidados intensivos, complicaciones clínicas y muerte por la enfermedad^22^.

En cuanto a la vacuna contra la COVID-19, los hallazgos de un estudio de metaanálisis señalan un efecto protector en personas mayores que recibieron el inmunizante. En un análisis con más de tres millones de participantes, las personas mayores vacunadas presentaron una reducción superior al 80% en las probabilidades de infección y muerte por COVID-19^23^. Asimismo, estudios recientes han señalado que la protección conferida por la vacunación en este grupo debe interpretarse a la luz de dos fenómenos complementarios: la inmunosenescencia y la disminución temporal de la efectividad vacunal, especialmente para los desenlaces de infección y enfermedad sintomática, con una preservación relativamente mayor frente a eventos graves. En este sentido, un metaanálisis internacional evidenció que la efectividad tiende a disminuir progresivamente con el paso de los meses y que las dosis de refuerzo restablecen niveles más elevados de protección, un elemento particularmente relevante para las personas mayores, en quienes la respuesta inmunitaria suele ser menos robusta y más heterogénea^24^. 

En consonancia, análisis realizados en redes asistenciales y de vigilancia de la efectividad en contextos reales han mostrado un beneficio adicional de vacunas actualizadas frente a variantes recientes, con un impacto más evidente en los grupos etarios de mayor edad. Estimaciones de efectividad en adultos mayores señalan una reducción importante de hospitalizaciones tras la vacunación estacional reciente, reforzando la necesidad de estrategias de refuerzo dirigidas a personas mayores y a grupos vulnerables, incluso en escenarios de predominio de sublinajes de Ómicron genéticamente divergentes de composiciones vacunales anteriores^25^ ^)-(^ ^27^. 

En cuanto al uso de soporte ventilatorio, las personas mayores hospitalizadas por COVID-19 admitidas en UCI presentan mayores probabilidades de muerte por la enfermedad cuando utilizan este recurso. Un estudio realizado con 1.994 participantes reveló que los individuos que utilizaron soporte ventilatorio no invasivo e invasivo presentaron un aumento de 2,25 y 24 veces, respectivamente, en las probabilidades de muerte por COVID-19 en comparación con aquellos que no necesitaron el dispositivo^28^. 

También es importante destacar que, además de la gravedad intrínseca del cuadro, la trayectoria de escalamiento del soporte ventilatorio puede influir en los desenlaces. En pacientes con insuficiencia respiratoria aguda por COVID-19, el uso de estrategias no invasivas (como la cánula nasal de alto flujo y la ventilación no invasiva) se ha asociado con beneficios en subgrupos seleccionados; sin embargo, las fallas en estas modalidades, cuando se acompañan de retraso en la intubación, pueden estar relacionadas con un peor pronóstico^29^. Estudios multicéntricos demostraron una mayor mortalidad hospitalaria en pacientes sometidos a intubación tardía en comparación con la intubación precoz, lo que refuerza la importancia de un monitoreo riguroso, criterios objetivos de fallo y una decisión clínica oportuna, especialmente en personas mayores, en quienes la reserva fisiológica es menor^30^. 

Además, la ventilación mecánica invasiva en este grupo específico se asocia con mayor frecuencia a desenlaces adversos relacionados con la hospitalización en UCI, como delirium, debilidad adquirida, prolongación de la sedación y complicaciones infecciosas, particularmente neumonía asociada a la ventilación mecánica, con impacto en la duración de la estancia hospitalaria y en la mortalidad^31^ ^)-(^ ^32^. Estudios con personas mayores en grupos etarios más avanzados también señalan peores resultados cuando se requiere ventilación no invasiva en contextos pandémicos, lo que sugiere que la edad avanzada, la fragilidad y la gravedad respiratoria tienden a sumarse como determinantes de una peor evolución clínica^33^. 

En el presente estudio se destacan algunas limitaciones inherentes al uso de datos secundarios provenientes de sistemas administrativos de salud, los cuales pueden contener subregistros o información incompleta en determinadas variables. No obstante, la metodología analítica adoptada fue cuidadosamente seleccionada para minimizar el impacto de datos faltantes en los análisis realizados, garantizando mayor rigor y confiabilidad en las inferencias relacionadas con la salud. Los resultados estadísticos que no asociaron comorbilidades preexistentes con la muerte por COVID-19 en este estudio pueden estar relacionados con la ausencia de un método de análisis estandarizado para investigaciones de esta naturaleza.

A pesar de estas limitaciones, los resultados obtenidos permitieron realizar un análisis detallado de las características epidemiológicas y clínicas de la infección y de la muerte por COVID-19 en personas mayores en Brasil. Además, se refuerza el papel de estudios con este tipo de diseño metodológico como impulsores de investigaciones similares en otras localidades, ampliando la comprensión sobre los determinantes contextuales y el comportamiento de la enfermedad en diferentes estratos poblacionales. Este enfoque resalta la relevancia de la producción científica orientada a la aplicación práctica y a la generación de beneficios concretos para la sociedad.

En este sentido, los hallazgos de este estudio se configuran como un insumo estratégico para los gestores de salud, al ofrecer evidencia científica para la formulación, cualificación e implementación de políticas públicas que reflejen la realidad experimentada por la población de personas mayores. Al considerar las vulnerabilidades existentes, tales políticas pueden promover intervenciones más resolutivas y eficaces, garantizando un impacto positivo en las acciones de salud y fortaleciendo la equidad y la calidad de la atención prestada.

## Conclusión

Los factores asociados a la muerte por COVID-19 en personas mayores hospitalizadas estuvieron relacionados con la edad, el sexo, la raza/color, la vacuna contra la COVID-19 y el uso de soporte ventilatorio. Además, tener 80 años o más, ser del sexo masculino, pertenecer a etnia indígena y haber utilizado soporte ventilatorio invasivo fueron condiciones asociadas a mayores probabilidades de muerte.

La administración de la vacuna contra la COVID-19 demostró ser eficaz al proporcionar protección a las personas mayores expuestas al SARS-CoV-2, evidenciando la importancia de las estrategias preventivas, como la inmunización, para minimizar los impactos adversos de la pandemia sobre un grupo poblacional más susceptible. Esta relación enfatiza la necesidad de mantener e intensificar las campañas de vacunación como estrategia de salud colectiva, con el fin de reducir las repercusiones multisistémicas de la COVID-19 en la vida, la salud y el bienestar de las personas mayores.

Se destaca la importancia de avanzar en las estrategias de monitoreo de la salud de las personas mayores, especialmente orientadas a ampliar la inmunoprevención y a disminuir la desinformación que rodea a las vacunas contra la COVID-19. Se espera que el presente estudio contribuya como base para el desarrollo de iniciativas que atiendan de manera integral las demandas específicas de la población de personas mayores. Entre estas iniciativas, se resalta el fortalecimiento de las acciones de salud en el nivel de atención primaria, con el objetivo de garantizar el acceso oportuno a los servicios de salud y la continuidad del cuidado, considerando las necesidades individuales de las personas mayores y buscando mitigar las vulnerabilidades de este grupo etario frente a la COVID-19.

## Data Availability

Todos los datos generados o analizados durante este estudio están incluidos en este artículo publicado.
